# Maternal plasma metabolic markers of neonatal adiposity and associated maternal characteristics: The GUSTO study

**DOI:** 10.1038/s41598-020-66026-5

**Published:** 2020-06-10

**Authors:** Ai-Ru Chia, Jamie V. de Seymour, Gerard Wong, Karolina Sulek, Ting-Li Han, Elizabeth J. McKenzie, Izzuddin M. Aris, Keith M. Godfrey, Fabian Yap, Kok Hian Tan, Lynette Pei-Chi Shek, Yung Seng Lee, Michael S. Kramer, Neerja Karnani, Mary Foong-Fong Chong, Philip N. Baker

**Affiliations:** 1 Department of Obstetrics & Gynaecology, Yong Loo Lin School of Medicine, National University of Singapore, Singapore, Singapore; 2 Saw Swee Hock School of Public Health, National University of Singapore, Singapore, Singapore; 3 School of Sport, Exercise and Nutrition, Massey University, Auckland, New Zealand; 4 Singapore Institute for Clinical Science, Agency for Science, Technology, and Research, Singapore, Singapore; 5 Liggins Institute, University of Auckland, Auckland, New Zealand; 6 Novo Nordisk Foundation Center for Protein Research, Faculty of Health and Medical Sciences, University of Copenhagen, Copenhagen, Denmark; 7 First Affiliated Hospital of Chongqing Medical University, Chongqing, China; 8 Division of Chronic Disease Research Across the Lifecourse, Department of Population Medicine Harvard Medical School and Harvard Pilgrim Health Care Institute, Boston, MA USA; 9 Medical Research Council Lifecourse Epidemiology Unit and National Institute for Health Research Southampton Biomedical Research Centre, University of Southampton and University Hospital Southampton National Health Service Foundation Trust, Southampton, United Kingdom; 10 Duke-NUS Medical School, Singapore, Nanyang Technological University, Singapore, Singapore; 11 Department of Pediatrics, KK Women’s and Children’s Hospital, Singapore, Singapore; 12 Lee Kong Chian School of Medicine, Nanyang Technological University, Singapore, Singapore; 13 Department of Reproductive Medicine, KK Women’s and Children’s Hospital, Singapore, Singapore; 14 Department of Pediatrics, Yong Loo Lin School of Medicine, National University of Singapore, Singapore, Singapore; 15 Khoo Teck Puat-National University Children’s Medical Institute, National University Health System, Singapore, Singapore; 16 Departments of Pediatrics and of Epidemiology, Biostatistics and Occupational Health, McGill University Faculty of Medicine, Montreal, Quebec Canada; 17 College of Life Sciences, University of Leicester, Leicester, UK

**Keywords:** Molecular biology, Paediatrics

## Abstract

Infant adiposity may be related to later metabolic health. Maternal metabolite profiling reflects both genetic and environmental influences and allows elucidation of metabolic pathways associated with infant adiposity. In this multi-ethnic Asian cohort, we aimed to (i) identify maternal plasma metabolites associated with infant adiposity and other birth outcomes and (ii) investigate the maternal characteristics associated with those metabolites. In 940 mother-offspring pairs, we performed gas chromatography-mass spectrometry and identified 134 metabolites in maternal fasting plasma at 26–28 weeks of gestation. At birth, neonatal triceps and subscapular skinfold thicknesses were measured by trained research personnel, while weight and length measures were abstracted from delivery records. Gestational age was estimated from first-trimester dating ultrasound. Associations were assessed by multivariable linear regression, with p-values corrected using the Benjamini-Hochberg approach. At a false discovery rate of 5%, we observed associations between 28 metabolites and neonatal sum of skinfold thicknesses (13 amino acid-related, 4 non-esterified fatty acids, 6 xenobiotics, and 5 unknown compounds). Few associations were observed with gestational duration, birth weight, or birth length. Maternal ethnicity, pre-pregnancy BMI, and diet quality during pregnancy had the strongest associations with the specific metabolome related to infant adiposity. Further studies are warranted to replicate our findings and to understand the underlying mechanisms.

## Introduction

Being born too early, too small, or too large is associated with impaired development and chronic diseases later in life^1–3^. Infants of similar weight often differ in adiposity^4^, and given the increasing trend of childhood obesity globally, investigation of infant adiposity is crucial in order to understand the early-life determinants of metabolic health^5^.

Metabolomics is the study of low molecular weight metabolites (<1000 Daltons)^6^ downstream from the omics cascade (genomics, transcriptomics, and proteomics) and most closely reflects the metabolic phenotype^7^. Maternal metabolite profiling is of interest and value in perinatology, because it provides a snapshot of the maternal physiological status. It therefore reflects both genetic and lifestyle (environmental) influences, thus allowing elucidation of metabolic pathways associated with birth outcomes^8–10^. While studies among non-pregnant populations have reported variations in metabolomic profiles associated with ethnicity, diet, adiposity, and smoking status^11–14^, it is unclear whether similar associations are observed during pregnancy, when the maternal body undergoes major physiological adaptions to support fetal development and growth^15^.

Several studies have demonstrated associations between maternal metabolite profiling during pregnancy and infant birth outcomes, based on biospecimens such as plasma^16–22^, urine^22–26^, amniotic fluid^26–28^, serum^29–32^, cervicovaginal fluid^33–35^, and hair^36^. Blood plasma is clinically accessible^37^ and potentially reflects processes in all organ systems. Compared to other biofluids, it is more comprehensive and contains the broadest array of metabolites^6^. Plasma metabolite profiles were typically analysed by liquid chromatography-mass spectrometry (LC-MS)^16,17,19,21^, flow injection analysis mass spectrometry (FIA-MS)^20^, or nuclear magnetic resonance (NMR)^18^, but none by gas chromatography-mass spectrometry (GC-MS), which has been regarded as the gold standard for metabolite profiling of volatile, low molecular weight metabolites due to its greater chromatographic resolution^8^.

In addition, most previous studies were conducted in Western countries^16–22^ and had sample sizes under 100 participants^17–19^, with minimal or no adjusment for confounders^16–18^. Larger multi-ethnic studies are therefore warranted. In our multiethnic Asian mother-offspring cohort study, we aimed to (i) identify maternal plasma metabolites associated with gestational duration and offspring birth size and adiposity and (ii) investigate the maternal characteristics associated with those metabolites to examine the potential sources of metabolic variation.

## Methods

### Study participants

First-trimester pregnant women aged 18–50 y were recruited from the National University Hospital and KK Women’s and Children’s Hospital between June 2009 and September 2010 to participate in the GUSTO (Growing Up in Singapore Towards healthy Outcomes) mother-offspring cohort^38^. Women were ineligible if their parents and spouses’ parents had different ethnicity (i.e., Chinese, Malay, or Indian descent), received chemotherapy or psychotropic drugs, or had type 1 diabetes. This study was approved by the National Health Care Group Domain Specific Review Board (reference D/09/021) and the SingHealth Centralized Institutional Review Board (reference 2009/280/D). All research was performed in accordance with the relevant guidelines and informed consent was obtained from all participants upon recruitment.

### Maternal metabolite assessment

#### Plasma sample preparation

We collected fasting blood samples at 26–28 week of gestation, processed within 4 h, and stored at −80 °C. Plasma samples were analysed in random order, and laboratory staff were blinded to birth outcomes. A 200 µL aliquot of plasma was spiked with two internal standard mixes: 20 µL of mix 1 prepared by dissolving 36 mg citric acid-d_4_, 34 mg alanine-d_4_, 26 mg tryptophan-d_5_, 23 mg hexanoic-d_11_ acid, 21 mg phenylalanine-d_5_, and 15 mg tyrosine-d_2_ in 36 ml of milli-Q water; and 20 µL of mix 2 prepared by dissolving 29 mg octanoic-d_15_ acid and 58 mg stearic-d_35_ acid in 36 ml of methanol. Quality control (QC) samples were prepared from a pool created from 40 µL aliquots of all samples. The samples were dried using a SpeedVac Concentrator (Thermo Scientific) at 80 Pa with no heating and stored at −80 °C.

Extraction of metabolites and derivatization (to increase compound volatility) were based on a published protocol^39^. Dried samples were suspended in 500 µL cold methanol-water solution (50% vol/vol), vortexed, centrifuged at 3500 rpm for 5 min at −4 °C, and the supernatant was collected. The same extraction process was repeated for methanol-water (80% vol/vol) and methanol solutions. The pooled supernatants were dried using a SpeedVac Concentrator and stored at −80 °C.

Dried samples were suspended in 400 µL sodium hydroxide (1 mol/L), 334 µL methanol and 68 µL pyridine in a silanized glass tube to reduce compound adherence to the glass wall (Thermo Scientific). The derivatization was initiated by adding 40 µL methyl chloroformate, followed by vigorous vortexing for 30 s. This process was repeated before adding 400 µL chloroform and 800 µL sodium bicarbonate (50 mmol/L), vortexing for 10 s after each addition. Derivatised samples were centrifuged at 2000 rpm for 3 min; the aqueous layer was removed and dehydrated with 300 mg anhydrous sodium sulphate before gas chromatography-mass spectrometry (GC-MS) analysis. Negative controls (without the addition of plasma and internal standards) underwent the same processing.

#### Gas chromatography-mass spectrometry (GC-MS) analysis

We analysed the samples on a GC-MS system—a GC7890B coupled to an MSD5977A operated at 70 eV electron ionization (Agilent Technologies)—equipped with a ZB-1701 column, 30 m × 0.25 mm × 0.15 μm with 5 m guard column (Phenomenex), at a constant flow rate of 1.0 mL/min of helium. Based on a published protocol^39^, samples (1 µL) were injected under pulsed splitless mode (for trace analyses) with the inlet at 290 °C (180 kPa for 1 min, 50 mL/min purge flow after 1 min). The GC oven temperature was initially held at 45 °C for 2 min, after which it was raised to 180 °C at 9 °C/min and held for 5 min. Subsequently, the temperature was raised to 220 °C at 40 °C/min and held for 5 min. The temperature was raised again to 240 °C at 40 °C/min and held for 11.5 min. Finally, the temperature was raised to 280 °C at 40 °C/min and held for 10 min. The interface was kept at 250 °C and the quadrupole at 130 °C. The detector operated in scan mode, started after 5.5 min with a mass range between 38–550 atomic mass units at 2.9 scans/s. Negative controls were injected at the beginning of each run and QCs were run every seventh sample.

#### Data processing

The GC-MS data were deconvoluted and metabolites were identified by the Automated Mass Spectral Deconvolution and Identification system software^40^ with the in-house methyl chloroformate^39^ and publicly available National Institute of Standards and Technology 14 mass spectra library^41^. We used the R Metab package for peak integration^42^. Features present in <5% of all samples and contaminants, identified by comparison with negative control samples, were excluded. Visual inspection of all extracted ion chromatograms was performed, and any integration errors were corrected. Each metabolite was normalised by one of the seven internal standards, chosen based on the highest linear correlation to the metabolite in the QC samples. All metabolites with an R^2^ < 0.75 with each and every internal standard were omitted from the analysis. We used median centering to align batches based on the correction factors derived from QCs in each of the batches^43^. Values below the limit of detection were assigned half of the minimum value for that metabolite^44^. We identified 134 metabolites; the median (IQR) coefficient of variation for all metabolites in the QC samples was 10.3 (8.2–13.4) and all were below the 30% threshold^45^. Metabolite values were log-transformed and centered before statistical analysis. Of the 134 metabolites, 43 were identified (80–100% match to a reference standard), 11 were putatively identified (80–100% mass spectral match), 38 were tentatively identified (60–79% mass spectral match), and 42 were unknown (<60% mass spectral match). We set the level of identification based on mass spectral matching levels and according to acceptable practices for chemical journals^46^.

### Infant characteristics

Gestational age (GA) was estimated by a dating scan in the first trimester; preterm birth was defined as delivery of a live birth <37 weeks of gestation. Birth weight was measured shortly after birth to the nearest 1 g (SECA 334; SECA Corp.), and recumbent length measured to the nearest 0.5 cm from the top of the head to the soles of the feet (SECA 210). Sex-specific birth weight-for-GA z-scores were derived using a global birth weight reference^47^ adapted for the GUSTO population^48^. Triceps and subscapular skinfold thicknesses, which have greater discriminative power than other anthropometric measurements for neonatal total body adiposity^49^, were measured in triplicate to the nearest 0.2 mm on the right side of the body by anthropometrists and summed (Holtain Skinfold Caliper; Holtain Ltd.). All research personnel were trained according to standardized procedures obtained from the PhenX toolkit^50^. Infant sex was retrieved from birth delivery reports.

### Maternal characteristics

Maternal age (in years, continuous), ethnicity (Chinese, Malay, or Indian), education (before secondary, postsecondary, or university), and self-reported pre-pregnancy weights were collected during recruitment. At 26–28 weeks’ gestation, maternal weight was measured to the nearest 0.1 kg (SECA 803) and standing height was measured in duplicate to the nearest 0.1 cm from the top of the head to the heels (SECA 213). Pre-pregnancy underweight (BMI < 18.5 kg/m^2^ or ≥18.5 kg/m^2^), pre-pregnancy overweight (BMI ≥ 23 kg/m^2^ or <23 kg/m^2^)^51^ was determined and weight gain until 26–28 weeks’ gestation (in kg, continuous) was calculated by subtracting pre-pregnancy weight from weight. Blood glucose levels were measured after fasting and two hours after a 75 g oral glucose load at 26–28 weeks’ gestation. Women were diagnosed with gestational diabetes (yes or no) if fasting glucose was ≥7.0 mmol/L and/or 2-hour post-glucose was ≥7.8 mmol/L according to the World Health Organization diagnostic criteria^52^. At 26–28 weeks’ gestation, information on diet quality during pregnancy (measured using the Healthy Eating Index for pregnant women in Singapore, continuous)^53^, physical activity (inactive, sufficiently active, or highly active)^54^, tobacco smoke exposure (plasma cotinine <0.17 ng/mL and no environmental tobacco smoke exposure, <0.17 ng/mL and self-reported environmental tobacco smoke exposure, 0.17–13.99 ng/mL, or ≥14 ng/mL)^55^, and alcohol consumption during pregnancy (yes or no) were also obtained. Parity (nulliparous or multiparous) and hypertensive disorders (yes or no) including chronic hypertension, pregnancy-induced hypertension, and pre-eclampsia were retrieved from delivery medical notes.

### Statistical analysis

Among the 1152 spontaneously conceived singleton pregnancies, 940 mothers who provided their blood samples and had metabolite data and information on their offspring birth outcomes were included in the analysis (Supplemental Fig. 1).

Maternal and child characteristics were summarized according to quintiles of gestational age, birth weight, birth length, and sum of neonatal skinfold thicknesses. P values for trend were assessed by modelling the median value of the quintiles in linear regression for continuous variables or Cochran-Mantel-Haenszel tests for categorical variables.

The associations of 134 maternal metabolites with offspring birth outcomes (i.e. gestational duration, birth weight, birth length, and sum of skinfold thicknesses) were assessed by multiple linear regression using a separate model for each metabolite. We conducted sensitivity analysis on sex-specific birth weight-for-GA z-scores to examine the robustness of our study results. The models were adjusted for infant sex and maternal age, parity, education, ethnicity, pre-pregnancy BMI, weight gain until 26–28 weeks’ gestation, height, tobacco smoke exposure, physical activity, diet quality, and gestational diabetes. The analysis approach is adopted and supported by recently published studies^21,31^. Metabolites associated with birth outcomes with a Benjamini-Hochberg false discovery rate (FDR) corrected q-value <0.10^56^ were selected for the next step of analysis^57^.

For the second aim of the study, relationships between maternal characteristics and those selected metabolites were independently examined using multiple linear regression. Metabolite z-scores were modelled as dependent variables while the independent variables were maternal characteristics. To derive individual estimates for each ethnic group, we created three variables: Chinese (yes or no), Malay (yes or no), and Indian (yes or no) and fitted the model with one ethnic variable at a time while adjusting for other maternal characteristics.

Missing data (height (*n* = 9), diet quality (*n* = 9), physical activity (*n* = 10), education (*n* = 11), alcohol use (*n* = 25), gestational diabetes (*n* = 36), pre-pregnancy BMI (*n* = 66), weight gain until 26–28 weeks of gestation (*n* = 73), and prenatal tobacco smoke exposure (*n* = 73)) were estimated by 20 imputations by the Markov chain Monte Carlo method; the reported results are those from the pooled analysis. To evaluate whether the imputation of missing data may have affected the results, we carried out a sensitivity analysis on participants with no missing data (*n* = 722).

We investigated potential effect modification by ethnicity by including an interaction term (ethnicity x metabolite) in the regression models. Ethnic differences were further explored by stratified analyses if the interaction term was significant (q-value <0.05). We performed sensitivity analyses to restrict analysis to term births (except for the analysis of gestational age). We also limited the analysis to women without hypertensive disorders, pre-eclampsia, or gestational diabetes (*n* = 536) in a separate sensitivity analysis.

All statistical analysis was performed in Stata 14 (StataCorp LP, USA). We considered q-values <0.05 statistically significant and 0.05 < q-values <0.10 as trends.

### Ethics approval and consent to participate

This study was approved by the National Health Care Group Domain Specific Review Board (reference D/09/021) and the SingHealth Centralized Institutional Review Board (reference 2009/280/D). All participants gave informed consent upon recruitment.

## Results

### Characteristics of participants and birth outcomes

This cohort comprised 55% Chinese, 26% Malays, and 19% Indians. The mean and standard deviation of maternal age was 30.5 ± 5.1 y, and 43% were overweight before pregnancy. Among the 940 infants, 7.6% were born preterm; the means and standard deviations of birth weight, birth length, and sums of neonatal skinfold thicknesses were 3094 ± 451 g, 48.6 ± 2.3 cm, and 10.3 ± 2.2 mm, respectively. Women with shorter gestational duration were more likely to have lower education, greater exposure to tobacco smoke during pregnancy, and hypertensive disorders. Heavier and longer infants were more likely to be born to mothers who were older, taller, had higher education, and had lower exposure to tobacco smoke. Infants with a lower sum of skinfold thicknesses were more likely to be born to mothers who were nulliparous and had higher education and higher diet quality. Mothers who were overweight before pregnancy and those of higher weight gain up to 26–28 weeks’ gestation were more likely to have infants who were heavier and have higher sum of skinfolds. Chinese infants were generally longer than Malay infants. Only results from the extreme quintiles are presented in Table 1 while all results are presented in Supplemental Table S1.

**Table 1:** Characteristics of participants according to quintiles of birth outcomes.

Characteristics	Gestational duration (week)			Birth weight (g)			Birth length (cm)			Sum of triceps and subscapular skinfold (mm)		
	Q1	Q5	P-trend	Q1	Q5	P-trend	Q1	Q5	P-trend	Q1	Q5	P-trend
	36 (37–38)	40 (40–40)		2580 (2383–2675)	3640 (3550–3805)		46 (46–47)	52 (52–53)		7.8 (7.3–8.2)	13.4 (12.6–14.8)	
Maternal age, y	30.3 ± 5.3	30.4 ± 4.9	0.84	29.9 ± 5.4	31.2 ± 4.5	0.01	30.0 ± 5.4	31.6 ± 4.3	0.002	29.9 ± 4.9	31.3 ± 5.1	0.02
Weight gain until 26–28 weeks, kg	9.2 ± 4.3	8.9 ± 4.6	0.64	7.9 ± 4.1	10.2 ± 4.7	<0.001	8.5 ± 4.9	8.8 ± 3.8	0.04	8.7 ± 4.6	9.9 ± 4.7	0.01
Height, cm	158 ± 5.8	159 ± 5.6	0.24	157 ± 5.4	159 ± 5.6	0.002	157 ± 5.7	159 ± 5.9	<0.001	159 ± 5.5	158 ± 5.5	0.08
HEI-SGP score	51 ± 13	53 ± 14	0.10	51 ± 13	52 ± 15	0.84	50 ± 12	57 ± 13	<0.001	54 ± 14	49 ± 13	<0.001
Ethnicity, %			0.23			0.23			0.05			0.54
Chinese	46	58		55	57		48	63		54	56	
Malay	35	20		23	27		33	16		27	32	
Indian	19	22		22	17		18	20		19	12	
Education, %			0.04			0.02			<0.001			0.005
None/primary/secondary	35	29		37	31		37	16		29	37	
Postsecondary	38	27		37	33		39	28		32	35	
University	28	44		26	36		24	56		39	28	
Physical activity, %			0.45			0.90			0.78			0.005
Inactive	27	31		32	31		30	26		38	28	
Sufficiently active	52	52		50	48		52	50		50	52	
Highly active	20	17		18	20		18	25		12	21	
Plasma cotinine, %			<0.001			0.002			<0.001			0.65
<0.17 ng/mL & no ETS exposure	43	68		45	58		41	70		51	48	
<0.17 ng/mL & self-reported ETS	31	19		36	30		35	21		27	34	
0.17–13.99 ng/mL	19	10		12	11		18	9.4		15	18	
≥14 ng/mL	7.0	2.3		7.9	1.7		7.0	0.0		7.9	0.6	
Pre-pregnancy overweight, %	44	42	0.89	37	50	0.002	44	45	0.92	37	57	0.003
Nulliparous, %	42	51	0.06	44	36	0.17	41	46	0.79	51	32	<0.001
Alcohol use during pregnancy, %	2.9	1.1	0.11	2.2	0.6	0.10	2.7	1.1	0.24	2.7	1.2	0.35
Gestational diabetes, %	23	17	0.13	19	23	0.80	17	28	0.06	14	22	0.43
Hypertensive disorders, %	8.1	1.6	<0.001	5.3	3.7	0.30	4.9	1.0	0.05	3.3	7.1	0.15
Male infant, %	58	52	0.26	48	64	<0.001	45	66	<0.001	60	37	<0.001

Only extreme quintiles are reported while all results are presented in Supplemental Table S1. Values are means ± SDs or medians (IQR). P-trends were assessed by modelling the median value of the quintiles in the linear regression analysis for continuous variables or Cochran-Mantel-Haenszel tests for categorical variables. There were missing data for pre-pregnancy BMI (*n *= 66), weight gain until 26–28 weeks (*n* = 73), height (*n* = 9), healthy eating index (*n* = 9), education (*n* = 11), physical activity (*n* = 10), plasma cotinine (*n* = 73), alcohol (*n* = 25), and gestational diabetes (*n* = 36). ETS, environmental tobacco smoke; HEI-SGP, Healthy Eating Index for pregnant women in Singapore; NA, not applicable; Q, quintile.

### Associations of maternal metabolites and birth outcomes

Only metabolites associated with birth outcomes (q-value <0.05) are presented in Table 2, while all results are presented in Supplemental Table S2. One metabolite was found to be inversely associated with gestational duration, and another metabolite with birth weight; they were tentatively identified as pyrazole, 3-nitro- and 10-nonadecenoic acid, methyl ester respectively. There were trends toward association of four metabolites and birth length, of which two were related to branched chain amino acids (3-methyl-2-oxopentanoic acid and isoleucine).

**Table 2:** Associations between maternal plasma metabolite levels and infant birth outcomes (q-value <0.05).

Metabolites	Metabolic sub-pathway	CAS number	Match (%)	β (95% CI)^1^	q-value
**Duration of gestation, weeks**					
Pyrazole, 3-nitro-	Chemical	26621–44–3	76	−0.81 (−1.24, −0.38)	0.03
**Birth weight, g**					
10-Nonadecenoic acid, methyl ester (C19:1)	Monounsaturated Fatty acid; Chemical	56599-83-8	66	−190 (−293, −88.0)	0.04
**Sum of triceps and subscapular skinfold thicknesses‘, mm**					
**Amino acid-related**					
Asparagine	Alanine & Aspartate	70-47-3	81	−1.17 (−1.74, −0.59)	<0.001
Creatinine	Creatine	60-27-5	93	−0.68 (−1.05, −0.30)	0.01
Methionine	Cysteine, Methionine, SAM, Taurine	63-68-3	97	−1.05 (−1.54, −0.55)	<0.001
Glutamine	Glutamate	56-85-9	81	−0.82 (−1.28, −0.36)	0.01
Glycine	Glycine, Serine, Threonine	56-40-6	87	−0.80 (−1.30, −0.31)	0.01
Serine	Glycine, Serine, Threonine	56-45-1	76	−1.14 (−1.71, −0.57)	<0.001
Threonine	Glycine, Serine, Threonine	72-19-5	93	−0.82 (−1.30, −0.34)	0.01
Lysine	Lysine	56-87-1	92	−0.91 (−1.49, −0.33)	0.02
Phenylalanine	Phenylalanine & Tyrosine	63-91-2	95	−1.10 (−1.73, −0.46)	0.01
Tyrosine	Phenylalanine & Tyrosine	60-18-4	92	−1.08 (−1.59, −0.57)	<0.001
Ornithine	Urea Cycle; Arginine & Proline	70-26-8	92	−0.55 (−0.94, −0.15)	0.03
Isoleucine	Valine, Leucine, Isoleucine	73-32-5	99	−0.68 (−1.05, −0.30)	0.01
Leucine	Valine, Leucine, Isoleucine	61-90-5	100	−0.73 (−1.13, −0.34)	0.01
**Lipids**					
2-Methyloctadecanoic acid	Branched Chain Fatty Acid; Chemical	7217-83-6	64	−0.73 (−1.22, −0.25)	0.02
Hexadecanoic acid, 14-methyl-, methyl ester	Branched Chain Fatty Acid; Chemical	2490-49-5	82	−0.46 (−0.80, −0.13)	0.03
Dodecanoic acid (C12:0)	Medium Chain Fatty Acid	143-07-7	94	−0.36 (−0.59, −0.13)	0.02
trans-Vaccenic acid OR Oleic acid OR cis-Vaccenic acid (C18:1)	Monounsaturated Fatty acid	693-72-1	79	−1.18 (−1.98, −0.39)	0.02
**Xenobiotics**					
2-Hydroxyisobutyric acid	Chemical	594-61-6	67	0.81 (0.19, 1.44)	0.05
5-Aminoimidazole-4-carboxylic acid, methyl ester	Chemical	4919-00-0	81	−1.13 (−1.79, −0.47)	0.01
Ethyl 2,5,8,11-tetraoxatridecan-13-oate	Chemical	91719382^*^	73	−0.75 (−1.26, −0.24)	0.02
glycine, N,N-bis(2-methoxy-2-oxoethyl)-, methyl ester	Chemical	22241-07-2	83	0.27 (0.18, 0.35)	<0.001
l-Leucine, N-methoxycarbonyl-, ethyl ester OR 2,2,2-trifluoroethyl ester	Chemical	88406-43-3	90	−0.41 (−0.71, −0.11)	0.03
**Unknown compounds**					
5-Cyano-4-methoxyamino-7-phenyl-hept-6-enoic acid, methyl ester	—	—	48	−0.81 (−1.32, −0.30)	0.02
5-Octadecenoic acid, methyl ester	—	—	58	−1.31 (−2.24, −0.38)	0.03
7-Benzofuranamine, 2-methyl-	—	—	49	−0.80 (−1.29, −0.32)	0.01
Benzene, hexyl-	—	—	59	−1.15 (−1.79, −0.50)	0.01
Imidazo[4,5-e][1,4]diazepin-8(1 H)-one, 4,5,6,7-tetrahydro-4,7-dimethyl-5-thioxo-	—	—	44	−0.74 (−1.27, −0.20)	0.04
Methylenedioxyamphetamine acetate	—	—	57	−0.91 (−1.47, −0.35)	0.01

β values are linear regression coefficients per SD increase in metabolite levels, adjusted for infant sex and maternal age, parity, education, ethnicity, pre-pregnancy BMI, weight gain until 26–28 weeks of gestation, height, physical activity, diet quality, plasma cotinine, and gestational diabetes. CAS, chemical abstracts service; SAM, s-adenosyl methionine. ^*^PubChem CID.

We observed inverse associations between 28 metabolites and infants’ sum of skinfold thicknesses at birth — 13 amino acid-related, 4 fatty acids, 6 xenobiotics, and 5 unknown compounds. Branched-chain fatty acids, medium-chain fatty acids (C10:0 and C12:0), and omega-6 fatty acids (C20:2 and C22:4) showed inverse trends with sum of skinfolds while 2-hydroxybutyric acid and 2-hydroisobutyric acid showed positive trends.

Potential interactions between ethnicity and metabolite associations for birth weight (5 metabolites) and birth length (8 metabolites) but not gestational age or sum of skinfolds (Supplemental Table S3). Stratified analyses were conducted, and higher branched-chain fatty acids were associated with higher birth weight and longer birth length in Indians but not in Chinese and Malays.

### Associations of maternal characteristics and infant adiposity-related metabolites

**Figure 1:**
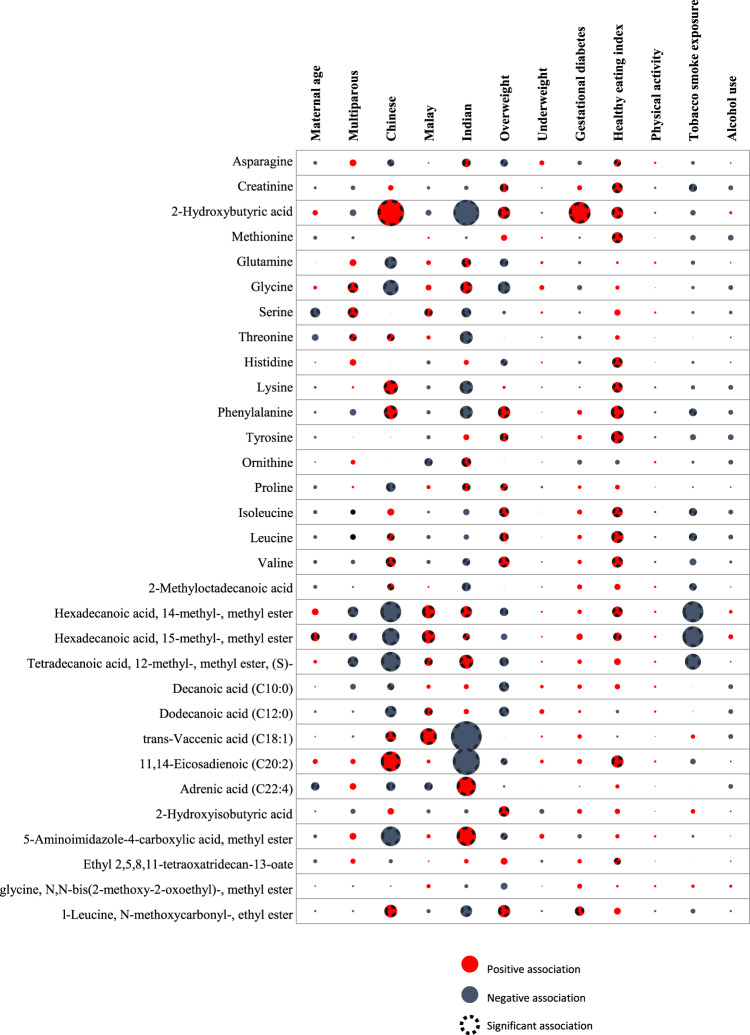
Maternal characteristics and infant adiposity-related metabolites. Multiple linear regression was performed with metabolite z-scores modelled as dependent variables and maternal characteristics as independent variables. Each circle denotes negative log-transformed q-values derived by the Benjamini-Hochberg procedure. Larger circles represent a lower q-value (i.e., a lower false discovery rate). (description for ‘Positive association’, ‘Negative association’ and ‘Significant association’).

Figure 1 summarizes the relationships between maternal characteristics and the infant adiposity-related metabolites. All results and estimates are presented in Supplemental Table S4. Ethnicity had the highest number of significant and independent associations with metabolites related to infant adiposity (*n* = 26). Generally, Chinese and Indian women showed contrasting associations. For example, levels of branched chain amino acids and 2-hydroxybutyric acid were higher in Chinese women but lower in Indian women. Metabolite profiles of Chinese women shared similarities with those of women who were overweight before pregnancy (*n* = 16 metabolites showed concordant direction of effect estimates).

Among the modifiable factors, pre-pregnancy overweight and maternal diet quality had the strongest associations with the metabolome (n = 17 and n = 15 metabolites, respectively). Few significant associations were found with parity, maternal age, tobacco smoke exposure during pregnancy, and gestational diabetes, and none with pre-pregnancy underweight, physical activity, and alcohol consumption during pregnancy.

Branched chain fatty acids were associated with most maternal factors. Lower levels were observed in participants who were exposed to tobacco smoke during pregnancy, multiparous, overweight, or of Chinese ethnicity, but higher levels were seen in Malay and Indian women and women with better diet quality.

### Sensitivity analysis

Results were largely consistent when we examined sex-specific birth weight-for-GA z-scores or adjusted for fasting or postprandial plasma glucose level instead of gestational diabetes. When we restricted our analysis of sum of skinfold thicknesses to participants with no missing data (*n* = 722), term births, or women without hypertensive disorders, or gestational diabetes (*n* = 536), results remained similar (data not shown).

## Discussion

In this multi-ethnic Asian cohort of 940 mother-offspring pairs, maternal levels of amino acid-related metabolites, non-esterified fatty acids, and xenobiotics were associated with neonatal sum of triceps and subscapular skinfold thicknesses. Few associations were observed with gestational duration, birth weight, or birth length. Maternal ethnicity, pre-pregnancy BMI, and diet quality during pregnancy had the strongest associations with the specific metabolome related to infant adiposity.

We are aware of one study that has examined the association between the maternal plasma metabolome and infant adiposity at birth^21^. In that United States study (*n* = 121), alkyl-linked phosphatidylcholines containing fatty acid 20:4 in the third trimester were associated with lower newborn body fat. However, differences in the analytical approach (targeted versus untargeted) and analytical platform (LC-MS versus GC-MS) limit our ability to compare our results to those of the previous study.

We observed an inverse association between amino acid-related metabolites and infant skinfold thickness. This finding is in line with our previous work, which showed higher maternal protein intake associated with lower abdominal internal adiposity in neonates^58^. However, we cautioned that protein intake does not necessarily mirror plasma concentrations of amino acids^59,60^ and that metabolite levels are also influenced by other maternal factors (e.g., ethnicity and pre-pregnancy overweight; see Fig. 1).

Congruent with previous studies, we showed positive trends between maternal 2-hydroxybutyrate levels and infant skinfold thickness. 2-hydroxybutyrate is a known early biomarker of insulin resistance^61^ and maternal glucose intolerance has also been shown (in our cohort and others) to be strongly associated with higher skinfold thickness in infancy^62,63^.

Accumulating evidence shows that elevated fatty acid concentrations are not necessarily associated with increased adiposity and insulin resistance^64^. Some fatty acids play a vital role in fetal growth and development^65^. In particular, medium chain fatty acids, which were associated with lower infant skinfold thickness in our study, have been shown to be related to higher oxidative metabolism and reduced adiposity in both animal and human studies^66^.

Branched-chain fatty acids (BCFA) showed inverse trends with infant skinfold thickness. We also observed lower levels of BCFA in women exposed to tobacco smoke during pregnancy, and in women who were multiparous, overweight, or of Chinese ethnicity, while higher levels were observed in Malay and Indian women and in women with better diet quality (Fig. 1). BCFA can be synthesized *de novo* or obtained from ruminant fats and milk^67^. Recent studies have reported that BCFA levels are positively associated with insulin sensitivity and are lower in obese than in lean persons^68,69^. However, inadequate information on lipid metabolism during pregnancy limits our understanding of the underlying mechanisms.

Several xenobiotics were significantly associated with the sum of skinfolds. However, their precise function and origin are largely unknown, except for 2-hydroxyisobutyric acid (2-HIBA). 2-HIBA is a gut microbiota-associated metabolite^70^ and may also reflect environmental exposure of the fuel additive, methyl tert-butyl ether^71^. Higher levels of 2-HIBA, which was associated with higher infant skinfold thickness in our study, have been observed in the urine of obese people^72^ and identified as a metabolic signature of adiposity^73^ and diabetes mellitus^74^.

We observed trends toward higher levels of branched-chain amino acid-related metabolites and longer birth length. This finding is in line with a cohort in United States examining cord blood metabolite patterns^75^. Pyrazole, 3-nitro- was found to be inversely associated with gestational duration, and 10-Nonadecenoic acid, methyl ester with birth weight. Based on their tentative identities, these metabolites have not been reported in earlier studies^16–19^, likely due to differences in analytical techniques (GC-MS versus LC-MS or NMR). We found associations between higher branched chain fatty acids and higher birth weight and longer birth length in Indians, but this was not observed in Chinese and Malays. Ethnic-specific associations have not been reported in previous studies.

Maternal ethnicity had the strongest associations with the specific metabolome related to infant adiposity. Chinese and Indian women often showed contrasting associations; these ethnic differences were also observed in our earlier work in which Chinese infants, compared to Indians, were more susceptible to excessive neonatal adiposity from high maternal glycemia^63^. We also observed that metabolite profiles of Chinese women were similar to women who were overweight even though Chinese women had a lower mean BMI than Malay and Indians in our study (mean ± SD: 21.6 ± 3.4 in Chinese; 24.2 ± 5.4 in Malay, 23.9 ± 4.5 in Indian). This may explain why the impact of increasing BMI on insulin resistance, C-reactive protein, and adiponectin levels is more pronounced in Chinese than in the other ethnic groups in Singapore^76^.

Among the modifiable factors, pre-pregnancy overweight and maternal diet quality were strongly associated with the metabolic signature of infant adiposity. Metabolite profiles have been shown to be reflective of diet quality^13^, but we did not observe similar diet-metabolite associations in our study. This difference may be attributable to differences in study population (pregnant versus non-pregnant)^13^ or in socio-cultural dietary habits. Congruent with our findings, pre-pregnancy BMI has been identified as an important driver of metabolic variation in a United States cohort^16^, and metabolites we found to be associated with overweight status have also been reported in other cohorts^14,66,68,77–79^. If our findings are replicated in other cohorts, formal mediation analysis would help sort out the degree to which the associations shown in Table 1 are mediated by the maternal metabolites or through other causal pathways

Strengths of our study include its prospective design (which minimizes interviewer and recall bias), the metabolomic profiling of a large multi-ethnic Asian population, the collection of lifestyle and clinical data (which permit adjustment for confounding factors), and the assessment of infant skinfold thicknesses, in addition to more common measures such as birth weight, which has been strongly associated with infant adipose stores and highlighted as a strong predictor of later child obesity risk^16^.

Several limitations of our study are worth noting. First, we have explored only the maternal plasma metabolome and concentrated on GC-MS analysis with methyl chloroformate derivatization. Although this method has optimized the detection of compounds with amino and/or carboxyl groups (which includes most metabolites of central carbon metabolism and key intermediates of cell metabolism^39^), our work could be complemented by employing different analytical techniques such as a variation of sample extraction methods or the use of multiple analytical platforms to obtain a more comprehensive overview of the metabolome. Second, we assessed maternal metabolites at 26–28 weeks of gestation, which precludes analysis of changes in metabolite levels across pregnancy. Third, we did not examine fetal growth throughout pregnancy; as a consequence, the temporal sequence between the maternal metabolome and infant birth size is unclear. Maternal metabolite profiles could have bi-directional relationships with fetal growth, such that abnormal fetal growth (e.g., intrauterine growth restriction) could influence maternal metabolite levels. Future studies should examine serial fetal biometry to address this issue. Last, as in any observational study, our findings could still be influenced by residual confounding such as imprecise measurements or underlying medical conditions despite the many potential confounding factors we adjusted for, and causality cannot be inferred.

In conclusion, we observed links between amino acid-related metabolites, non-esterified fatty acids, and infant adiposity at birth measured by sum of skinfold thickness. Few associations were observed with gestational duration, birth weight, or birth length. Maternal ethnicity, pre-pregnancy BMI, and diet quality had the strongest associations with the metabolome. Further independent studies are warranted to replicate our findings and to understand the underlying mechanisms.

## Supplementary information

Supplementary information.

Supplementary information2.

## Data Availability

Data are available upon request because data request for analysis must be assessed and approved by the executive committee of the GUSTO study and administered by the main GUSTO study team of data management, which is a third-party source. Any matter related to data request, please contact Mary Chong Foong Fong at mary_chong@nus.edu.sg
