# Supplementary material for: Maternal plasma metabolic markers of neonatal adiposity and associated maternal characteristics: The GUSTO study
Source: Sci Rep. 2020 Jun 10;10:9422. doi: 10.1038/s41598-020-66026-5 (PMC7287081; doi:10.1038/s41598-020-66026-5)
Supplement: Supplementary file 2 — Supplementary information2. [file 41598_2020_66026_MOESM2_ESM.docx]

**Supplemental Figure 1**

Flowchart showing selection of participants included in this analysis from GUSTO (Growing Up in Singapore Towards healthy Outcomes) study, Singapore.

| Recruited  *n* = 1247 |  |  |
| --- | --- | --- |
|  |  | Excluded  *n* = 95  In vitro fertilization (*n* = 85)  Multiple pregnancy (*n* = 10) |
| Eligible  *n* = 1152 |  |  |
|  |  | Dropout  *n* = 64  Lost to follow-up  Family disapproval  Personal reasons  Inconvenience |
|  |  |  |
|  |  | Excluded  *n* = 148  Did not have metabolites data |
| Included for analysis  *n* = 940 |  |  |
